# Supplementary material for: DMRforPairs: identifying Differentially Methylated Regions between unique samples using array based methylation profiles
Source: BMC Bioinformatics. 2014 May 15;15:141. doi: 10.1186/1471-2105-15-141 (PMC4046028; doi:10.1186/1471-2105-15-141)
Supplement: Additional file 2 — DMRforPairs output for the comparison of A431-MCF7 and NA17018-NA17105. Please start from the HTML files in each folder. Available via the BMC Bioinformatics website. [file 1471-2105-15-141-S2.zip › 1394847754114233_MOESM2_ESM/A431_MCF7/figures/34728.pdf]

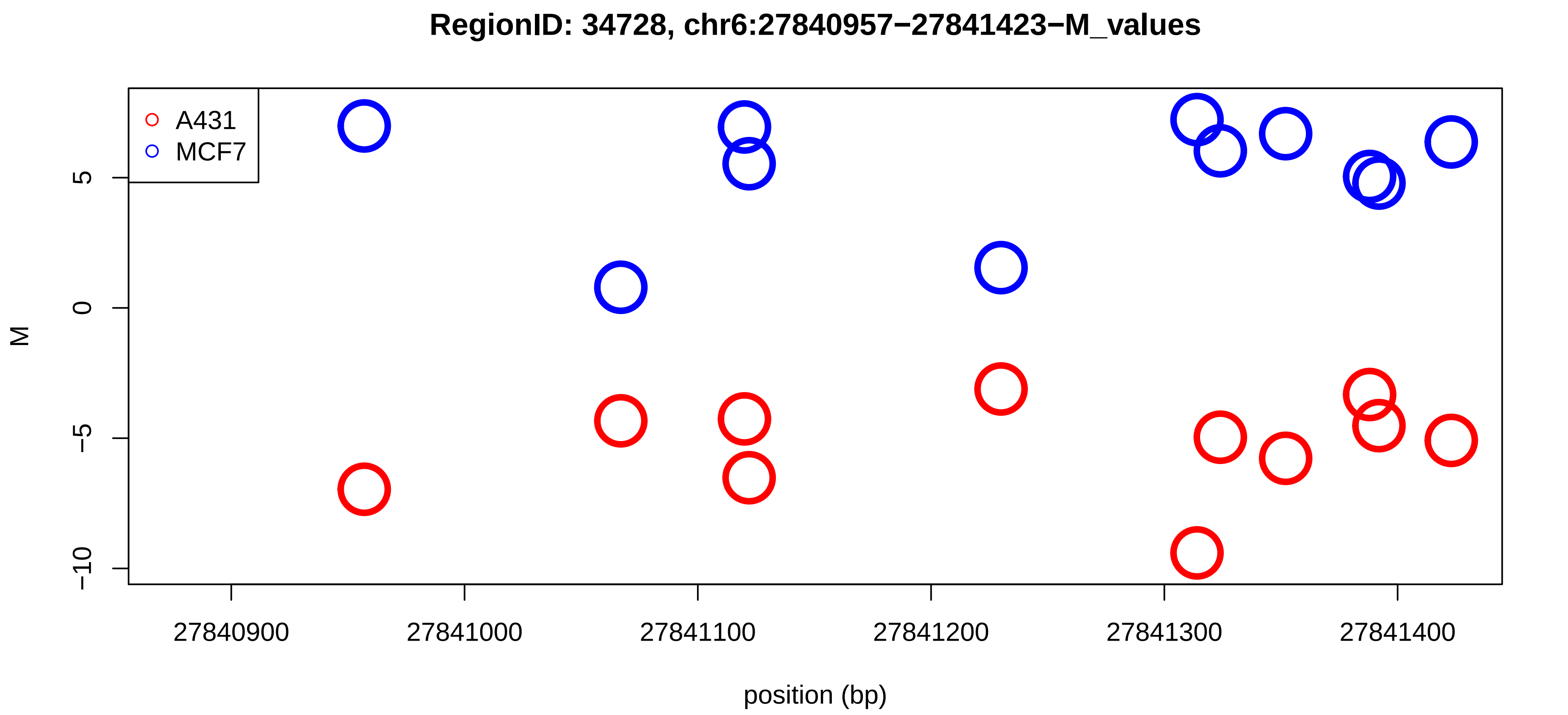

RegionID: 34728, chr6:27840957−27841423−M_values
A431
MCF7
M
5
0
−5
−10
27840900
27841000
27841100
27841200
27841300
27841400
position (bp)

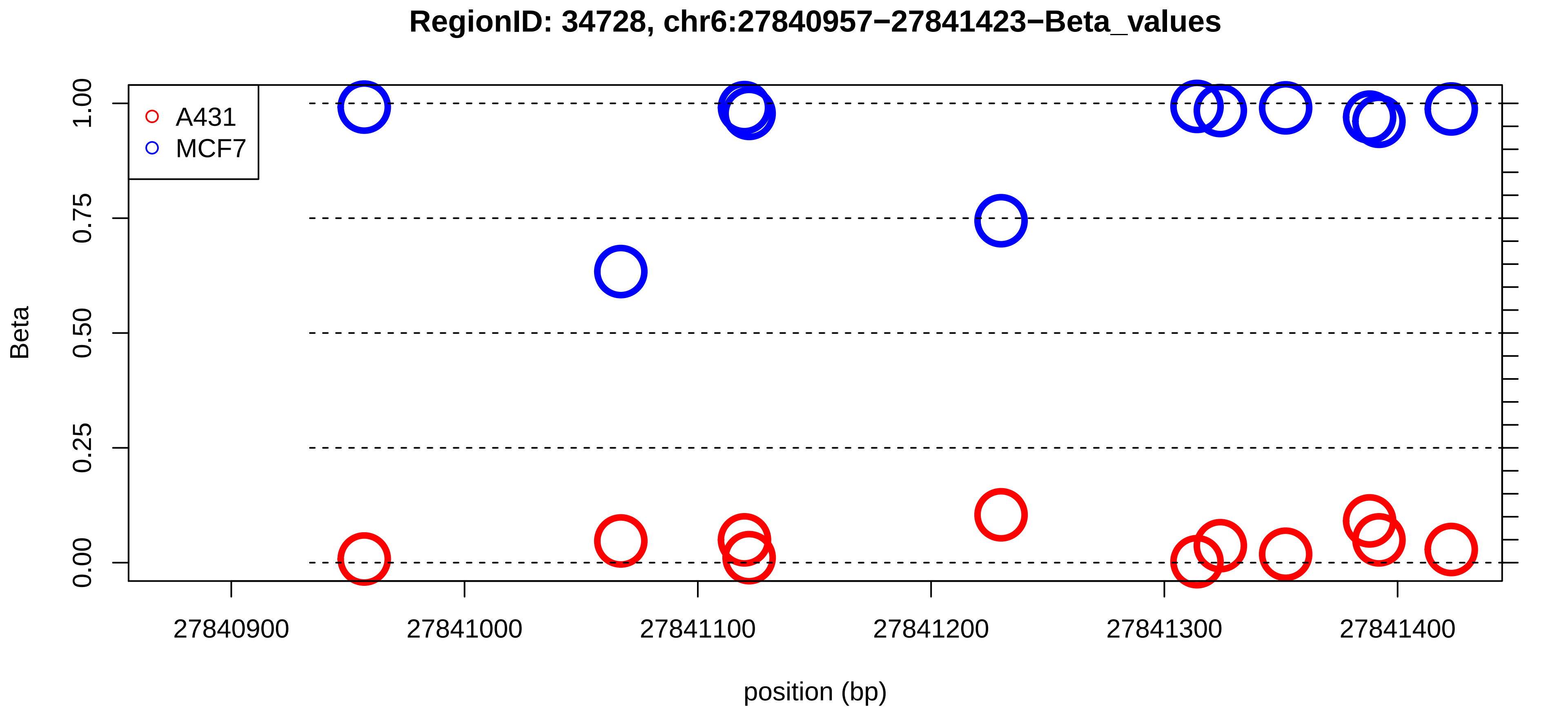

RegionID: 34728, chr6:27840957−27841423−Beta_values
A431
MCF7
Beta
1.00
0.75
0.50
0.25
0.00
27840900
27841000
27841100
27841200
27841300
27841400
position (bp)

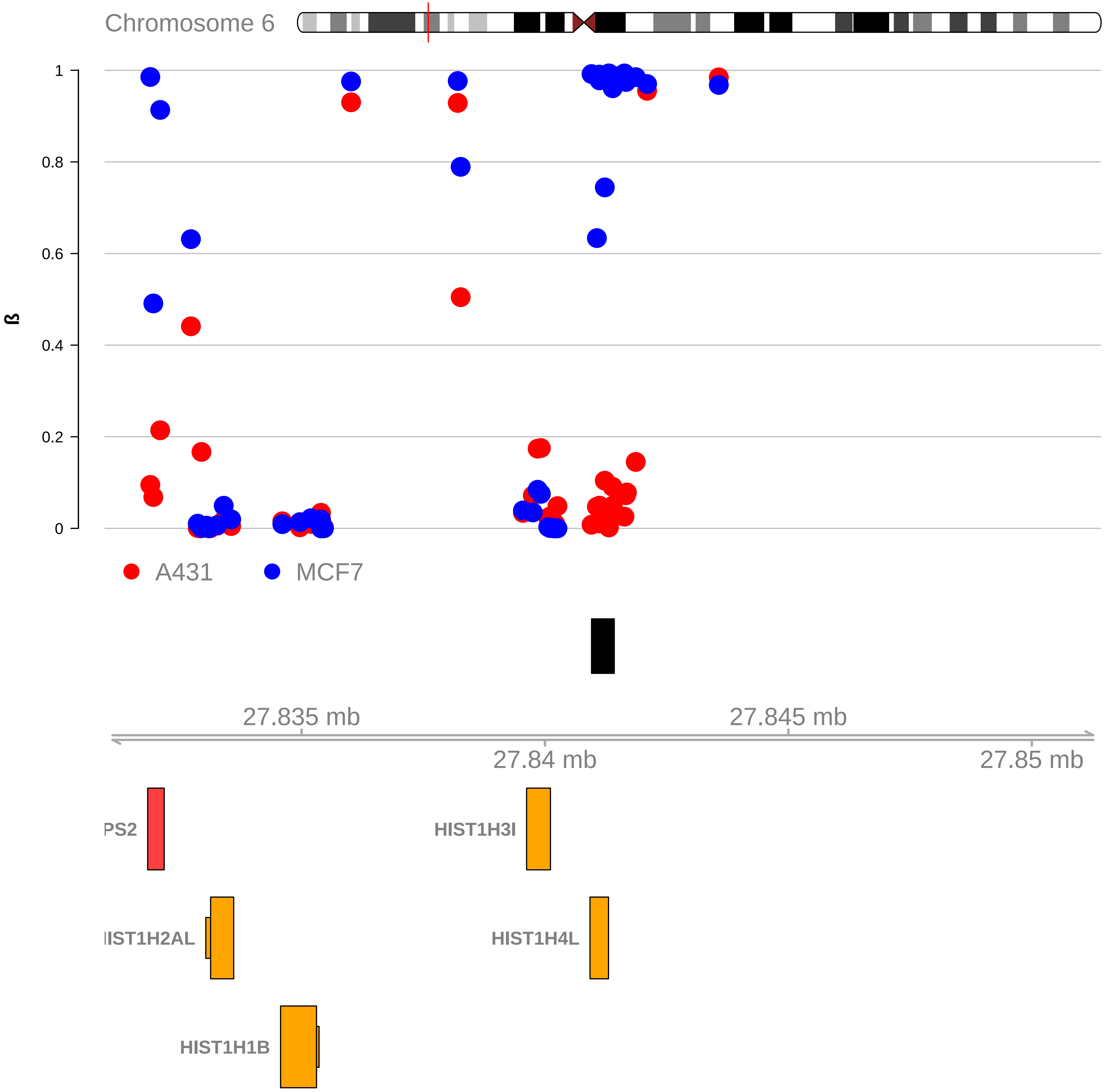
Chromosome 6
ß
1
0.8
0.6
0.4
0.2
0
A431
MCF7
27.835 mb
27.845 mb
27.84 mb
27.85 mb
PS2
HIST1H3I
IIST1H2AL
HIST1H4L
HIST1H1B
